# Prevalence and factors associated with admission to the intensive care unit in children hospitalized for community-acquired pneumonia

**DOI:** 10.17843/rpmesp.2023.404.12872

**Published:** 2023-12-18

**Authors:** Melany Mosquera-Rojas, Jenny Rondón-Saldaña, Patricia Llaque-Quiroz

**Affiliations:** 1 Universidad Peruana de Ciencias Aplicadas, Lima, Peru. Universidad Peruana de Ciencias Aplicadas Universidad Peruana de Ciencias Aplicadas Lima Peru; 2 Instituto Nacional de Salud del Niño San Borja, Lima, Peru. Instituto Nacional de Salud del Niño San Borja Lima Peru

**Keywords:** Pneumonia, Intensive Care, Children, Patient Admission

## Abstract

**Objective.:**

To determine the prevalence and factors associated with intensive care unit admission in children and adolescents with community-acquired pneumonia.

**Materials and methods.:**

Analytical cross-sectional observational study at the Instituto Nacional de Salud del Niño San Borja in 2019. The sample consisted of children older than one month and younger than 18 years who were admitted to emergency diagnosed with community-acquired pneumonia. We used Poisson regression to assess association.

**Results.:**

We evaluated 166 patients diagnosed with pneumonia, 94 (56.6%) were male and the median age was 24 months (IQR: 11 - 48). Most patients had a mild modified PIRO score of 136 (81.9%); 31 (18.7%) patients had complicated pneumonia and 24 (14.5%) were admitted to intensive care. The higher the age, the lower the prevalence of admission to ICU (PR=0.99, 95%CI: 0.98-0.99). The severity assessed with the modified PIRO score (PR=3.40, 95%CI: 1.46-7.93) and the presence of complicated pneumonia (PR: 5.88, 95%CI: 2.46-14.06) were associated with admission to intensive care.

**Conclusions.:**

The prevalence of admission to intensive care in children with community-acquired pneumonia was 14.5%. Younger patients with pneumonia, with greater severity assessed with the modified PIRO score and with complicated pneumonia have a higher prevalence of admission to intensive care.

## INTRODUCTION

Pneumonia is the leading cause of death of infectious origin in children worldwide and accounts for 22% of all deaths in children between 1 and 5 years of age ^1^. In Peru, the incidence of pneumonia in children under five years of age is 10.2 cases per 10,000 inhabitants and has a case fatality rate of 0.9 ^2^.

Scales are used to assess the severity of pneumonia in adults, and the use of these scales has been shown to reduce the frequency of hospitalization and administration of broad-spectrum antibiotics ^3^. However, there are no validated criteria for assessing the severity of community-acquired pneumonia in children. The World Health Organization (WHO) defines severe pneumonia as a case in which the child is unable to drink, has persistent vomiting, convulsions, lethargy, stridor or severe malnutrition; however, these criteria have high sensitivity but low specificity ^4^. A multicenter cohort study developed a predictive model for severe outcomes such as death, need for mechanical ventilation or use of vasoactive agents in children hospitalized with pneumonia; it showed that age extremes, altered vital signs, presence of retractions and radiographic infiltrates were the most important predictive factors ^5^. However, these results cannot be extrapolated to emergency patients and their clinical usefulness is still unknown.

Scales have been designed to predict mortality in children with pneumonia such as the modified PIRO scale (modified Predisposition, Insult, Response and Organ dysfunction), the RISC scale (Respiratory Index of Severity in Children) and the PRESS scale (Pediatric Respiratory Severity Score). The modified PIRO and RISC scales are good predictors of mortality due to pneumonia in children. The PIRO scale has a greater capacity to discriminate mortality and could be used in more complex health centers ^6^. However, these scales have not been evaluated to predict admission to the ICU (intensive care unit) in children with pneumonia.

Early recognition of the factors associated with adverse outcomes and higher prevalence of admission to intensive care in children with pneumonia in emergency areas is important because it allows timely actions to be taken that can reduce mortality in this age group. Therefore, the aim of this study is to determine the prevalence of admission to ICU and its associated factors in children and adolescents hospitalized with pneumonia at the Instituto Nacional de Salud del Niño San Borja (INSNSB) in 2019.

**KEY MESSAGES**

Motivation for the study: Pneumonia is an important cause of admission to intensive care in children. Having tools to recognize which children are most at risk for admission to ICU is important for emergency physicians.

Main findings: Patients with younger age, higher severity assessed with the modified PIRO score and with complicated pneumonia have a higher prevalence of admission to intensive care.

Implications: Emergency assessment of objective parameters such as the modified PIRO score may allow decisions to be made regarding the admission of children with pneumonia.

## MATERIALS AND METHODS

### Study design

Analytical cross-sectional study.

### Setting

The study was carried out at the INSNSB, a healthcare center located in Lima, Peru, which is a national referral institute for the care of children with complex surgical pathologies. This institution has five intensive care units specialized in the management of patients with cardiac, cardiovascular, neurosurgical, burn and general diseases ^7^.

### Participants

The population consisted of children and adolescents with a diagnosis of community-acquired pneumonia hospitalized at INSNSB during 2019. A census-type sampling was carried out. We included patients older than one month and younger than 18 years; patients with primary or secondary immunodeficiency and those with chronic lung disease such as cystic fibrosis, primary ciliary dyskinesia or bronchopulmonary dysplasia were excluded.

### Procedures

The medical records of patients hospitalized with the diagnosis of community-acquired pneumonia during 2019 identified with ICD-10 codes J18.0 to J18.9 were requested from the INSNSB statistics area. Compliance with study selection criteria was then assessed through review of the medical records. The epidemiological and clinical variables were obtained from the information in the medical records; the laboratory and imaging variables were taken from the SISGALENPLUS and Pacs programs, respectively. The SISGALENPLUS program stores the laboratory results ^8^ and the Pacs program stores the images and radiological reports of the patients seen at the INSNSB.

### Variables

The outcome variable “admission to the intensive care unit” was assessed according to the medical record (yes/no). The decision to admit the patient to the ICU is made by the physicians in charge of the patient. Admission criteria are found in hospital guidelines; however, they are different according to each ICU.

Variables such as prematurity, presence of comorbidities and congenital malformations were obtained from the medical record (yes/no). In addition, respiratory distress (yes/no) was considered to be the presence of any of the signs such as nasal flaring, whining, nodding or use of accessory muscles according to the report on admission of the patient. A patient was considered to have complicated pneumonia if any of the following diagnoses were described in the medical record: pleural effusion, empyema, lung abscess, necrotizing pneumonia, pneumothorax or bronchopleural fistula (yes/no). The assessment of multilobar involvement was performed by reviewing the radiographic report in the Pacs program on the radiograph at hospital admission; the patient was considered as having this condition if two or more lobes were involved. Tachycardia and tachypnea were considered when the heart rate and respiratory rate, respectively, were above the 95th percentile for age according to the study by O'leary *et al*. ^9^. Variables such as C-reactive protein levels, procalcitonin and neutrophil-lymphocyte index (NLI) are numerical variables and were taken from the Galenhos system with recording of the value closest to hospital admission. The modified PIRO score, calculated according to clinical history data within 48 hours of emergency admission, was classified as mild (0-2 points), moderate (3-4 points), high (5-6 points) or very high (7-10 points) according to the score obtained by adding one point for each of the following criteria: age less than six months or comorbidities, hypoxia (oxygen saturation <90%), hypotension according to age, complicated or multilobar pneumonia and multiorgan dysfunction (presence of renal failure, acute respiratory distress syndrome and acute liver failure) ^10^.

### Statistical analysis

The database was stored in Excel and then exported to the statistical package Stata v.15.0 (StataCorp, TX, US). Qualitative variables were described with absolute and relative frequencies and quantitative variables with median and interquartile range. The bivariate analysis of the categorical variables was performed with Pearson’s Chi-square test if less than 20% of the expected values were less than 5; if this assumption was not met, Fisher’s exact test was used. The quantitative variables were analyzed with the Mann Whitney U test because the assumptions of normality and homogeneity of variances were met. The measure of association was the prevalence ratio, measured with raw values and adjusted with 95% confidence intervals. A generalized linear model (GLM) of the Poisson family of robust variance was used after evaluating the statistical assumptions for this model, such as the independence of the observations and the response variable being a count per unit of time. The variables that were statistically significant (p<0.05) in the bivariate analysis were included in the adjusted model. The “presence of comorbidities” variable was not included in the adjusted model because it is a criterion for assessing the severity of the PIRO score, so there is collinearity between the two variables. No collinearity was found between the variables age and complicated pneumonia according to the variance inflation factor, which was less than 10.

### Ethical aspects

This study was approved by the Ethics Committee of the Universidad Peruana de Ciencias Aplicadas (PI272-19) and the INSNSB (PI-412). A waiver of informed consent was requested because this was a retrospective study that analyzed medical records without direct contact with patients. The data from the medical records of the study participants were kept anonymously by the researchers in a database created for the study.

## RESULTS

### Population selection

We identified 245 patients with a diagnosis of community-acquired pneumonia hospitalized at INSNSB during 2019. Of these, the medical records of 72 patients were not found on file and seven patients were excluded due to eligibility criteria, five patients with secondary immunodeficiency and two patients with chronic lung disease. Finally, the sample consisted of 166 patients and 24 (14.5%) of them were admitted to the intensive care unit.

### Epidemiological, clinical and laboratory characteristics

Most patients were male (n=94, 56.6%) and the median age was 24 months (IQR: 11-48). Regarding medical history, 29 patients (17.5%) had been premature, one third had congenital cardiac malformation (31.3%) and 33 (19.9%) had comorbidities, the most common being Down syndrome.

On admission to the emergency room, 76 patients (45.8%) presented tachypnea and almost all had respiratory distress (n=158, 95.2%). Only 10.2% (17/98) of blood cultures were positive. Most had a mild modified PIRO score (n=136, 81.9%). The median neutrophil-lymphocyte index was 2.3 (IQR: 1.2-4.2). Of the patients, 36.1% had multilobar involvement and 18.7% had complicated pneumonia (Table 1).

**Table 1:** Epidemiological, clinical and laboratory characteristics of patients hospitalized for community-acquired pneumonia at the Instituto Nacional de Salud del Niño San Borja, 2019 (n=166).

Variables	Patients (%)
Male sex	94 (56.6)
Age (months) ^a^	24 (11-48)
Weight (kg) ^a^	10.7 (7.5-14.8)
Place of origin	
Lima	105 (63.2)
Provinces	61 (36.8)
Prematurity	29 (17.5)
Congenital cardiac malformation	52 (31.3)
Comorbidities	33 (19.9)
Respiratory distress	158 (95.2)
Tachycardia	43 (25.9)
Tachypnea	76 (45.8)
Modified PIRO Score	
Mild	136 (81.9)
Moderate	26 (15.7)
High	4 (2.4)
Oxygen saturation (%) ^a^	95 (92-97)
C-reactive protein (mg/L) ^a^	24 (8-59.2)
Procalcitonin (ng/mL) ^a^	0.3 (0.08-1)
Neutrophil-lymphocyte index ^a^	2.3 (1.2-4.2)
Blood culture	
Does not have	68 (41)
Negative	81 (48.8)
Positive	17 (10.2)
Multilobar involvement in radiography	60 (36.1)
Complicated pneumonia	31 (18.7)
Time of hospitalization (days)^a^	10 (7-16)
Admission to ICU	24 (14.5)

a Median (interquartile range)

ICU: Intensive Care Unit, PIRO: (P) Predisposition, (I) Insult, (R) Respond and (O) Organ dysfunction.

### Bivariate analysis of admission to intensive care

The bivariate analysis showed that procalcitonin levels were higher in patients admitted to ICU (0.5 vs. 0.17; p=0.033) and the duration of hospitalization in days was longer in patients who were admitted to ICU when compared to those who were not (17.5 vs. 10, p<0.001). Patients with comorbidities were 2.87 times more likely to be admitted to ICU with respect to patients without comorbidities (p=0.004, 95%CI: 1.4-5.9) (Table 2 and Table 3).

**Table 2:** Association of intensive care unit admission with clinical, epidemiological and laboratory characteristics in children hospitalized for community-acquired pneumonia at the Instituto Nacional de Salud del Niño San Borja, 2019 (n=166).

Variables	Admission to UCI		p- value
	Yes n=24 (%)	No n=142 (%)	
Sex			
Male	16 (66.7)	78 (54.9)	0.283 ^a^
Female	8 (33.3)	64 (45.0)	
Age (months) ^c^	12 (9-36)	24 (12-48)	0.184
Weight (kg) ^c^	10.6 (7.4-15.6)	10.7(1.5-15.0)	0.287
Prematurity			
Yes	33 (12.5)	26 (18.3)	0.771^b^
No	21 (87.5)	116 (81.7)	
Cardiac malformation			
Yes	9 (37.5)	43 (30.3)	0.481 ^a^
No	15 (62.5)	99 (69.7)	
Comorbidities			
Yes	10 (41.7)	23 (16.2)	0.004 ^a^
No	14 (58.3)	119 (83.8)	
Sensorium alteration			
Yes	20 (83.3)	120 (84.5)	0.542 ^b^
No	4 (16.7)	22 (15.5)	
Respiratory Distress			
Yes	22 (91.7)	136 (95.8)	0.326 ^b^
No	2 (8.3)	6 (4.2)	
Tachycardia			
Yes	5 (20.8)	38 (26.8)	0.623 ^b^
No	19 (79.2)	104 (73.2)	
Tachypnea			
Yes	9 (37.5)	67 (47.2)	0.379 ^a^
No	15 (62.5)	75 (52.8)	
Modified PIRO Score			
Mild	8 (33.3)	128 (90.1)	<0.001 ^b^
Moderate	12 (50.0)	14 (9.9)	
High	4 (16.7)	0 (0)	
Oxygen saturation (%) ^c^	95 (92.5-98.0)	95 (92-97)	0.686
C-reactive protein (mg/L) ^c^	30.4 (3.7-59.2)	24 (8.7-58.9)	0.938
Procalcitonin (ng/mL) ^c^	0.5 (0.2-1.9)	0.17 (0.1-0.9)	0.033
Neutrophil-lymphocyte index ^c^	3.1 (1.4-3.8)	2.2 (1.2-4.2)	0.299
Blood culture			
Does not have	5 (20.8)	63 (44.4)	0.013 ^b^
Positive	6 (25.0)	11 (7.7)	
Negative	13 (54.2)	68 (47.9)	
Multilobar involvement in radiography			
Yes	12 (50.0)	48 (33.8)	0.127**
No	12 (50.0)	94 (66.2)	
Complicated pneumonia			
Yes	17 (70.8)	14 (9.9)	<0.001 ^a^
No	7 (29.2)	128 (90.1)	
Time of hospitalization (días) ^c^	17.5 (12.5-31.5)	10 (7-15)	<0.001

We used the Mann Whitney U test to evaluate the association between numerical variables and the outcome variable. ^a^ Chi-square test, ^b^ Fisher's exact test, ^c^ median (interquartile range). ICU: intensive care unit, PIRO: (P) Predisposition, (I) Insult, (R) Respond and (O) Organ dysfunction.

**Table 3:** Crude and adjusted multivariate analysis of intensive care unit admission in children with community-acquired pneumonia at the Instituto Nacional de Salud del Niño San Borja, 2019 (n=166).

Variable	Admission to ICU (cPR)	95%Ci	p- value	Admission to ICU (aPR)	95%Ci	p- value ^a^
Sex						
Female	0.65	0.29-1.44	0.292			
Male	Ref.					
Age (months)	0.98	0.97-0.99	0.042	0.99	0.98-0.99	0.013
Weight (kg)	0.95	0.90-1.00	0.053			
Place of origin						
Lima	Ref					
Province	1.22	0.58-2.60	0.589			
Prematurity						
Yes	0.67	0.21-2.12	0.501			
No	Ref.					
Cardiac malformation						
Yes	1.31	0.61-2.81	0.480			
No	Ref.					
Comorbidities						
Yes	2.87	1.40-5.9	0.004			
No	Ref.					
Sensorium alteration						
Yes	0.92	0.34-2.50	0.884			
No	Ref.					
Respiratory distress						
Yes	0.55	0.15-1.97	0.365			
No	Ref.					
Tachycardia						
Yes	0.75	0.29-1.89	0.547			
No	Ref.					
Tachypnea						
Yes	0.71	0.33-1.53	0.385			
No	Ref.					
Modified PIRO Score						
Mild	Ref.			Ref.		
Moderate	7.84	3.55-17.33	<0.001	3.40	1.46-7.93	0.004
High	17.00	8.66-33.36	<0.001	4.33	1.86-10.09	0.001
Oxygen saturation	0.99	0.90-1.09	0.906			
C-reactive protein (mg/L)	1.00	0.99-1.00	0.616			
Procalcitonin (ng/ml)	1.01	0.98-1.04	0.389			
Neutrophil/lymphocyte ratio	1.03	0.91-1.18	0.554			
Blood culture						
Negative	Ref.					
Positive	2.19	0.97-4.97	0.058			
Does not have	0.46	0.17-1.22	0.120			
Multilobar involvement in radiography						
Yes	1.76	0.84-3.69	0.130			
No	Ref.					
Complicated pneumonia						
Yes	10.57	4.79-23.33	<0.001	5.88	2.46-14.06	<0.001
No	Ref.			Ref.		

a We adjusted for age, modified PIRO score and complicated pneumonia according to statistical criteria. The comorbidities variable was not included in the multivariate analysis because it is a parameter included in the modified PIRO score and therefore there is collinearity.

cPR: crude prevalence ratio, aPR: adjusted prevalence ratio, ICU: intensive care unit.

PIRO: (P) Predisposition, (I) Insult, (R) Respond and (O) Organ dysfunction.

### Multivariate analysis of admission to intensive care

The multivariate analysis showed that for each additional month of life, the probability of ICU admission decreases by 1% (PR: 0.99, p=0.011, 95%CI: 0.98-0.99). Patients with complicated pneumonia were five times more likely to be admitted to ICU (PR=5.88, p<0.001, 95%CI: 2.46-14.06) than patients with uncomplicated pneumonia. Likewise, patients with moderate modified PIRO score had 3.4 times the probability of ICU admission of those with mild modified PIRO score (PR=3.40, p=0.004, 95%CI:1.46-7.93) and patients with high modified PIRO score had 4.33 times the probability of ICU admission compared to those with mild modified PIRO score (PR=4.33, p=0.001, 95%CI:1.86- 10.09). A statistically significant association was found in all cases even after adjusting for variables such as age and complicated pneumonia (Table 3).

## DISCUSSION

In this study, we reviewed the medical records of 166 hospitalized patients diagnosed with community-acquired pneumonia at INSNSB. The prevalence of admission to the ICU was 14.5%. Younger age, having complicated pneumonia and higher severity assessed with the modified PIRO score were variables associated with admission to intensive care.

The prevalence of admission to ICU in this study (14.5%) was lower than that reported by Fritz *et al*., who studied children under 18 years of age hospitalized for pneumonia in three hospitals in the United States enrolled in the EPIC study (Etiology of Pneumonia in the Community Study), and found that 21% of those without bacteremia and 43% of those with bacteremia were admitted to intensive care ^11^. However, the prevalence of admission to intensive care was lower (8%) in a prospective study of children under five years of age admitted for pneumonia in a hospital in Vietnam ^12^. Results have been similar to ours in reports from other Latin American countries; a cross-sectional study in children under five years of age aimed at identifying predisposing factors for severe pneumonia in children hospitalized in a Colombian hospital found a prevalence of admission to ICU of 11.3% ^13^. These differences may be due to several factors, the level of complexity of the hospitals included in the study, the age of the patients included and the criteria for admission to the ICU in each one.

This study found that when children with pneumonia are younger, the prevalence of admission to intensive care in is higher. Similarly, other studies have found an association between younger patients age and higher prevalence of adverse outcomes such as mortality. Thus, Djelantik *et al*. described that patients younger than four months had higher mortality compared to older children (RR=3.5, 95%CI: 3.0-4.2) ^14^. These results are explained by anatomical and physiological factors that are more unfavorable at a younger age, such as the absence of interalveolar and alveolo-bronchiolar ventilation channels that favor the appearance of complications such as atelectasis, greater airway resistance, lower pulmonary compliance and greater compliance of the thoracic cage in children under two years of age, and the immaturity of the innate and acquired immune system.

Complicated pneumonia (parapneumonic effusion, empyema) was associated with a higher probability of admission to intensive care. Results similar to those of Golbart *et al*., who reported that the presence of empyema is associated with admission to the ICU ^15^. It has also been reported that the presence of moderate or large pleural effusion is associated with admission to the ICU in children (OR: 3.2, 95%CI: 1.1-8.9) ^16^. Space-occupying conditions in the pleura can lead to respiratory failure due to hypoventilation, which may explain the greater need for ventilatory support in the ICU in these patients.

The modified PIRO score discriminates the probability of dying in children hospitalized with pneumonia and may be a reliable tool for selecting patients requiring admission to intensive care ^17^^,^^18^. This study found that patients with moderate and high modified PIRO scores were more likely to be admitted to the ICU than patients with mild PIRO scores. These results could be an indication of the usefulness of this score in predicting admission to the ICU; however, we did not find information in the medical records for all the items considered in this score, such as the blood pressure value.

This study found no association between multilobar involvement on chest radiography and ICU admission. Unlike Williams DJ *et al*., who reported that multilobar infiltrates were associated with severe adverse outcomes such as ICU admission, mechanical ventilation and death ^5^. These discordant results could be due to factors that may alter the interpretation of chest radiograph findings such as hydration status, presence of atelectasis, and time of presentation ^19^. For our study, we selected patients who were in the emergency area at hospital admission.

No association was found between INL levels and admission to the ICU. This index was assessed with the first hemogram on admission, a time in which the patient probably had not received antibiotics yet. This result is different from those reported by Lee *et al*., who found that elevated INL was associated with greater mortality and was a predictor of admission to the ICU in adults; this difference could be due to the immune development of children, in whom cellular immunity predominates over innate immunity, which could explain why they have a greater number of lymphocytes than neutrophils compared to adults ^20^.

Patients with comorbidities were more likely to be admitted to the ICU only in the crude model. This is consistent with the results published by Koh *et al*. who found that the presence of comorbidities is an early prognostic variable for unfavorable outcomes in patients admitted to the intensive care unit ^21^.

This study has limitations. Since this was a retrospective study, some variables of interest have not been evaluated, such as vaccination status against pneumonia-causing pathogens, nutritional status or the time the patient spent in the emergency room before going to the ICU. In addition, the blood pressure value was not available for all patients and the alteration of this value is used to construct the modified PIRO score. There are no unified criteria for deciding admission to the ICU in children with pneumonia in the INSNSB. In addition, the sample size is small so there are wide confidence intervals and there could be real associations not found in the present study.

In conclusion, the prevalence of admission to intensive care in children with pneumonia was 14.5%. Younger age, the presence of complicated pneumonia and greater severity assessed with the modified PIRO score were associated with admission to the ICU in children with community-acquired pneumonia. It is recommended that modified PIRO be applied to predict admission to the ICU in prospective studies with larger sample sizes to improve the accuracy of the results.
