# Supplementary material for: Prevalence and factors associated with admission to the intensive care unit in children hospitalized for community-acquired pneumonia
Source: Rev Peru Med Exp Salud Publica. 2023 Dec 18;40(4):406–12. [Article in Spanish] doi: 10.17843/rpmesp.2023.404.12872 (PMC11138820; doi:10.17843/rpmesp.2023.404.12872)
Supplement: Translation

# Translation: Prevalencia y factores asociados al ingreso a la unidad de cuidados intensivos en niños hospitalizados por neumonía adquirida en la comunidad

**Authors:** Melany Mosquera-Rojas, Jenny Rondón-Saldaña, Patricia Llaque-Quiroz

**Affiliations:** 1 Universidad Peruana de Ciencias Aplicadas, Lima, Perú.; 2 Instituto Nacional de Salud del Niño San Borja, Lima, Perú.

**Keywords:** Neumonía, Unidad de Cuidados Intensivos Pediátricos, Niños, Admisión del Paciente

## Abstract

**Objetivo.:**

Determinar la prevalencia y los factores asociados al ingreso a la unidad de cuidados intensivos en niños y adolescentes con neumonía adquirida en la comunidad.

**Materiales y métodos.:**

Estudio observacional transversal analítico en el Instituto Nacional de Salud del Niño San Borja en el 2019, la muestra estuvo conformada por niños de mayores de un mes y menores de 18 años que ingresaron a emergencia con diagnóstico de neumonía adquirida en la comunidad. Se utilizó la regresión de Poisson para evaluar asociación.

**Resultados.:**

Se evaluaron 166 pacientes con diagnóstico de neumonía, 94 (56,6%) fueron varones y la mediana de la edad fue 24 meses (RIC: 11‒48). La mayoría de los pacientes presentó un puntaje PIRO modificado leve de 136 (81,9%), 31 (18,7%) pacientes tuvieron neumonía complicada y 24 (14,5%) ingresaron a cuidados intensivos. A mayor edad se halló menor prevalencia de ingreso a UCI (RP=0,99, IC95%: 0,98‒0,99); la gravedad evaluada con el score PIRO modificado (RP=3,40, IC95%: 1,46‒7,93) y la presencia de neumonía complicada (RP: 5,88, IC95%: 2,46‒14,06) estuvieron asociados al ingreso a cuidados intensivos.

**Conclusiones.:**

En niños con neumonía adquirida en la comunidad la prevalencia de ingreso a cuidados intensivos fue de 14,5%. Los pacientes con neumonía de menor edad, con mayor gravedad evaluada con el puntaje PIRO modificado y con neumonía complicada tienen mayor prevalencia de ingreso a cuidados intensivos.

## INTRODUCCIÓN

La neumonía es la principal causa de muerte de origen infeccioso en niños de todo el mundo y constituye la causa del 22% de todas las muertes de niños entre 1 a 5 años ^1^. En el Perú, la incidencia de neumonía en niños menores de cinco años es de 10,2 casos por cada 10 000 habitantes y tiene una letalidad de 0,9 ^2^.

Se han descrito escalas para evaluar la gravedad de neumonía en adultos, el uso de estas escalas ha demostrado reducir la frecuencia de hospitalización y administración de antibióticos de amplio espectro ^3^. Sin embargo, no existen criterios validados para evaluar la gravedad de la neumonía adquirida en la comunidad en niños. La Organización Mundial de la Salud (OMS) define a neumonía grave como aquella en la que el niño no puede beber, tiene vómitos persistentes, convulsiones, letargo, estridor o malnutrición grave; sin embargo, estos criterios tienen una alta sensibilidad, pero baja especificidad ^4^. A través de un estudio multicéntrico de cohortes se desarrolló un modelo de predicción para resultados graves como muerte, necesidad de ventilación mecánica o uso de vasoactivos en niños hospitalizados con neumonía; se encontró que los extremos de edad, la alteración de los signos vitales, la presencia de retracciones y los infiltrados radiográficos fueron los factores predictivos más importantes ^5^. Sin embargo, estos resultados no pueden ser extrapolados a pacientes atendidos en emergencia y su utilidad clínica aún se desconoce.

Se han diseñado escalas para predecir mortalidad en niños con neumonía como la escala PIRO modificado (Predisposition, Insult, Response and Organ dysfunction modificado), la escala RISC (Respiratory Index of Severity in Children) y la escala PRESS (Pediatric Respiratory Severity Score). Las escalas PIRO modificado y RISC son buenos predictores de mortalidad por neumonía en niños. La primera escala tiene mayor capacidad discriminatoria de mortalidad y podría ser usada en centros de salud de mayor complejidad ^6^. Sin embargo, estas escalas no se han valorado para predecir ingreso a UCI (unidad de cuidados intensivos) en niños con neumonía.

Es importante el reconocimiento temprano en áreas de emergencia de los factores que se asocian a resultados adversos y mayor prevalencia de ingreso a cuidados intensivos en niños con neumonía debido a que permite tomar acciones oportunas que pueden disminuir la mortalidad en este grupo. Por lo tanto, el objetivo de este estudio es determinar la prevalencia de ingreso a UCI y sus factores asociados en niños y adolescentes hospitalizados con neumonía en el Instituto Nacional de Salud del Niño San Borja (INSNSB) en el 2019.

**MENSAJE CLAVE**

Motivación para realizar el estudio: La neumonía es una causa importante de ingreso a cuidados intensivos en niños. Tener herramientas para reconocer qué niños tienen más riesgo de ingreso a estas unidades es importante para los médicos en emergencia.

Principales hallazgos: Los pacientes con menor edad, mayor gravedad evaluada con el score PIRO modificado y con neumonía complicada tienen mayor prevalencia de ingreso en cuidados intensivos.

Implicancias: La evaluación en emergencia de parámetros objetivos como el score PIRO modificado pueden permitir tomar decisiones con respecto al ingreso de niños con neumonía.

## MATERIALES Y MÉTODOS

### Diseño del estudio

Estudio transversal analítico.

### Entorno

El estudio se ha llevado a cabo en el INSNSB, centro ubicado en Lima, Perú y que constituye un instituto de referencia nacional que tiene el objetivo de atender niños con patologías quirúrgicas complejas. Esta institución cuenta con cinco unidades de cuidados intensivos especializadas en el manejo de pacientes con patología cardiaca, cardiovascular, neuroquirúrgica, quemados y general ^7^.

### Participantes

La población estuvo conformada por niños y adolescentes con diagnóstico de neumonía adquirida en la comunidad, hospitalizados en el INSNSB durante el 2019. Se realizó un muestreo tipo censo. Se incluyeron a los pacientes mayores de un mes y menores de 18 años; se excluyeron a los pacientes con inmunodeficiencia primaria o secundaria y aquellos con enfermedad pulmonar crónica como fibrosis quística, discinesia ciliar primaria o displasia broncopulmonar.

### Procedimientos

Se solicitó al área de estadística del INSNSB el número de las historias clínicas de los pacientes hospitalizados con el diagnóstico de neumonía adquirida en la comunidad durante el 2019 identificado con códigos de CIE-10 de J18,0 al J18,9. Luego se evaluó el cumplimiento de criterios de selección del estudio a través de la revisión de las historias clínicas. Las variables epidemiológicas y clínicas fueron obtenidas a partir de la información consignada en las historias clínicas; las variables laboratoriales e imagenológicas fueron tomadas de los programas SISGALENPLUS y Pacs respectivamente. El programa SISGALENPLUS almacena los resultados de laboratorio ^8^ y el programa Pacs las imágenes e informes radiológicos de los pacientes atendidos en el INSNSB.

### Variables

La variable resultado «ingreso a la unidad de cuidados intensivos» fue evaluada según el registro en la historia clínica (sí/no). La decisición de ingreso a UCI es tomada por los médicos a cargo del paciente. Existen guías hospitalarias con criterios de ingreso, sin embargo, son diferentes de acuerdo con cada UCI.

Las variables como prematuridad, presencia de comorbilidades y malformaciones congénitas se obtuvieron de los datos de la historia clínica (sí/no). Además, se consideró como dificultad respiratoria (sí/no) a la presencia de alguno de los signos como aleteo nasal, quejido, cabeceo o uso de músculos accesorios según el reporte al ingreso del paciente. Respecto a la variable neumonía complicada, se consideró presente si alguno de los siguientes diagnósticos estaba descrito en la historia clínica: efusión pleural, empiema, absceso pulmonar, neumonía necrotizante, neumotórax o fístula broncopleural (sí/no). La valoración del compromiso multilobar se realizó mediante la revisión del informe radiográfico en el programa Pacs sobre la radiografía al ingreso hospitalario, se consideró presente si dos o más lóbulos estaban comprometidos. Se consideró taquicardia y taquipnea cuando la frecuencia cardíaca y frecuencia respiratoria, respectivamente, estaban por encima del percentil 95 para la edad según el estudio de O’leary *et al*. ^9^. Las variables como niveles de proteína C reactiva, procalcitonina e índice neutrófilos-linfocitos (INL) son variables numéricas y se tomaron del sistema Galenhos con registro del valor más cercano al ingreso hospitalario. El score PIRO modificado, calculado según los datos de la historia clínica dentro de las 48 horas al ingreso a emergencia, fue clasificado como leve (0-2 puntos), moderado (3-4 puntos), alto (5-6 puntos) o muy alto (7-10 puntos) según el puntaje obtenido al sumar un punto por cada uno de los siguientes criterios presentes: edad menor a seis meses o comorbilidades, hipoxia (saturación de oxígeno <90%), hipotensión de acuerdo a la edad, neumonía complicada o multilobar y disfunción multiorgánica (presencia de falla renal, síndrome de distrés respiratorio agudo y falla hepática aguda) ^10^.

### Análisis estadístico

La base de datos se registró en Excel y luego se exportó al paquete estadístico Stata v.15.0 (StataCorp, TX, US). Las variables cualitativas fueron descritas con frecuencias absolutas y relativas y las variables cuantitativas con mediana y rango intercuartílico. El análisis bivariado de las variables categóricas se realizó con la prueba de Chi cuadrado de Pearson si menos del 20% de los valores esperados fueron menores a 5, de no cumplirse este supuesto, se utilizó la prueba exacta de Fisher; y de las variables cuantitativas con la prueba de U de Mann Whitney debido a que se cumplieron los supuestos de normalidad y homogeneidad de varianzas. La medida de asociación fue la razón de prevalencia, medida con valores crudos y ajustados con intervalos de confianza del 95%, se usó un modelo lineal generalizado (GLM) de la familia Poisson de varianza robusta luego de evaluar los supuestos estadísticos para este modelo como son la independencia de las observaciones y la variable de respuesta es un recuento por unidad de tiempo. Para el ingreso de variables al modelo ajustado, se tomó en cuenta el criterio estadístico, ingresaron al modelo ajustado las variables que tuvieron significancia estadística (p<0,05) en el análisis bivariado. La variable «presencia de comorbilidades» no ingresó en el modelo ajustado debido a que es un criterio para evaluar la gravedad del score PIRO por lo que hay colinealidad entre ambas variables. No se encontró colinealidad entre las variables edad y neumonía complicada según el factor de inflación de varianza que fue menor de 10.

### Aspectos éticos

El presente estudio fue aprobado por el Comité de Ética de la Universidad Peruana de Ciencias Aplicadas (PI272-19) y del INSNSB (PI-412). Se solicitó exención del consentimiento informado debido a que es un estudio retrospectivo con revisión de historias clínicas sin contacto alguno con pacientes. Los datos de las historias clínicas de los participantes del estudio son resguardados en forma anónima por los investigadores en una base de datos creada para el estudio.

## RESULTADOS

### Selección de población

Se identificaron 245 pacientes con diagnóstico de neumonía adquirida en la comunidad hospitalizados en el INSNSB durante el 2019. De ellos, no se encontraron las historias clínicas de 72 pacientes en archivo y se excluyeron siete pacientes por criterios de elegibilidad, cinco pacientes con inmunodeficiencia secundaria y dos pacientes con enfermedad pulmonar crónica. Finalmente, la muestra estuvo conformada por 166 pacientes y 24 (14,5%) de ellos ingresaron a la unidad de cuidados intensivos.

### Características epidemiológicas, clínicas y laboratoriales

La mayoría de los pacientes fueron varones (n=94, 56,6%) y la mediana de la edad fue 24 meses (RIC: 11-48). Con respecto a los antecedentes, 29 pacientes (17,5%) habían sido prematuros, la tercera parte tenía alguna malformación cardiaca congénita (31,3%) y 33 (19,9%) presentaban comorbilidades, la más común fue síndrome de Down.

Al ingreso a emergencia, 76 pacientes (45,8%) presentaron taquipnea y casi todos, dificultad respiratoria (n=158, 95,2%). Solo el 10,2% (17/98) de los hemocultivos fue positivo. La mayoría presentó un score PIRO modificado leve (n=136, 81,9%). La mediana del índice neutrófilos-linfocitos fue 2,3 (RIC: 1,2‒4,2). El 36,1% de los pacientes tenía compromiso multilobar y 18,7% neumonía complicada (Tabla 1).

**Tabla 1:** Características epidemiológicas, clínicas y laboratoriales de pacientes hospitalizados por neumonía adquirida en la comunidad en el Instituto Nacional de Salud del Niño San Borja, 2019 (n=166).

Variables	Pacientes (%)
Sexo masculino	94 (56,6)
Edad (meses) ^a^	24 (11-48)
Peso (kg) ^a^	10,7 (7,5-14,8)
Lugar de procedencia	
Lima	105 (63,2)
Provincia	61 (36,8)
Prematuridad	29 (17,5)
Malformación cardiaca congénita	52 (31,3)
Comorbilidades	33 (19,9)
Dificultad respiratoria	158 (95,2)
Taquicardia	43 (25,9)
Taquipnea	76 (45,8)
Score PIRO modificado	
Leve	136 (81,9)
Moderado	26 (15,7)
Alto	4 (2,4)
Saturación de oxígeno (%) ^a^	95 (92-97)
Proteína C reactiva (mg/L) ^a^	24 (8-59,2)
Procalcitonina (ng/mL) ^a^	0,3 (0,08-1)
Índice neutrófilos linfocitos ^a^	2,3 (1,2-4,2)
Hemocultivo	
No tiene	68 (41)
Negativo	81 (48,8)
Positivo	17 (10,2)
Compromiso multilobar en radiografía	60 (36,1)
Neumonía complicada	31 (18,7)
Tiempo de hospitalización(días)^a^	10 (7-16)
Ingreso a UCI	24 (14,5)

a Mediana (rango intercuartil)

UCI: unidad de cuidados intensivos, PIRO: (P) Predisposition, (I) Insult, (R) Respond and (O) Organ dysfunction

### Análisis bivariado de ingreso a cuidados intensivos

En el análisis bivariado se encontró que los niveles de procalcitonina fueron más altos en los pacientes que ingresaron a UCI (0,5 vs. 0,17; p=0,033) y la duración de la hospitalización en días fue mayor en los pacientes que ingresaron a UCI con respecto con los que no lo hicieron (17,5 vs. 10, p<0,001). Los pacientes con comorbilidades tuvieron 2,87 veces la probabilidad de ingreso a UCI con respecto a los pacientes sin comorbilidades (p=0,004, IC95%: 1,4‒5,9) (Tabla 2 y Tabla 3).

**Tabla 2:** Asociación de ingreso a unidad de cuidados intensivos con características clínicas, epidemiológicas y laboratoriales en niños hospitalizados por neumonía adquirida en la comunidad en el Instituto Nacional de Salud del Niño San Borja, 2019 (n=166).

Variables	Ingreso a UCI		Valor de p
	Sí n=24 (%)	No n=142 (%)	
Sexo			
Masculino	16 (66,7)	78 (54,9)	0,283 ^a^
Femenino	8 (33,3)	64 (45,0)	
Edad(meses) ^c^	12 (9-36)	24 (12-48)	0,184
Peso(kg) ^c^	10,6 (7,4-15,6)	10,7(1,5-15,0)	0,287
Prematuridad			
Sí	33 (12,5)	26 (18,3)	0,771^b^
No	21 (87,5)	116 (81,7)	
Malformación cardiaca			
Sí	9 (37,5)	43 (30,3)	0,481 ^a^
No	15 (62,5)	99 (69,7)	
Comorbilidades			
Sí	10 (41,7)	23 (16,2)	0,004 ^a^
No	14 (58,3)	119 (83,8)	
Alteración de sensorio			
Sí	20 (83,3)	120 (84,5)	0,542 ^b^
No	4 (16,7)	22 (15,5)	
Dificultad Respiratoria			
Sí	22 (91,7)	136 (95,8)	0,326 ^b^
No	2 (8,3)	6 (4,2)	
Taquicardia			
Sí	5 (20,8)	38 (26,8)	0,623 ^b^
No	19 (79,2)	104 (73,2)	
Taquipnea			
Sí	9 (37,5)	67 (47,2)	0,379 ^a^
No	15 (62,5)	75 (52,8)	
Score PIRO modificado			
Leve	8 (33,3)	128 (90,1)	<0,001 ^b^
Moderado	12 (50,0)	14 (9,9)	
Alto	4 (16,7)	0 (0)	
Saturación de oxígeno (%) ^c^	95 (92,5-98,0)	95 (92-97)	0,686
Proteína C reativa (mg/L) ^c^	30,4 (3,7-59,2)	24 (8,7-58,9)	0,938
Procalcitonina (ng/mL) ^c^	0,5 (0,2-1,9)	0,17 (0,1-0,9)	0,033
Índice neutrófilos linfocitos ^c^	3,1 (1,4-3,8)	2,2 (1,2-4,2)	0,299
Hemocultivo			
No tiene	5 (20,8)	63 (44,4)	0,013 ^b^
Positivo	6 (25,0)	11 (7,7)	
Negativo	13 (54,2)	68 (47,9)	
Compromiso multilobar en radiografía			
Sí	12 (50,0)	48 (33,8)	0,127**
No	12 (50,0)	94 (66,2)	
Neumonía complicada			
Sí	17 (70,8)	14 (9,9)	<0,001 ^a^
No	7 (29,2)	128 (90,1)	
Tiempo de hospitalización (días) ^c^	17,5 (12,5-31,5)	10 (7-15)	<0,001

Para evaluar la asociación entre variables numéricas y la variable resultado se usó la prueba de U de Mann Whitney.^a^ Prueba de Chi cuadrado, ^b^ prueba exacta de Fisher, ^c^ mediana (rango intercuartílico).UCI: unidad de cuidados intensivos, PIRO: (P) Predisposition, (I) Insult, (R) Re-spond and (O) Organ dysfunction.

**Tabla 3:** Análisis multivariado crudo y ajustado de ingreso a la unidad de cuidados intensivos en niños con neumonía adquirida en la comunidad en el Instituto Nacional de Salud del Niño San Borja, 2019 (n=166).

Variable	Ingreso a UCI (RPc)	IC95%	Valor de p	Ingreso a UCI (RPa)	IC95%	Valor de p ^a^
Sexo						
Femenino	0,65	0,29-1,44	0,292			
Masculino	Ref.					
Edad(meses)	0,98	0,97-0,99	0,042	0,99	0,98-0,99	0,013
Peso(kg)	0,95	0,90-1,00	0,053			
Lugar de procedencia						
Lima	Ref					
Provincia	1,22	0,58-2,60	0,589			
Prematuridad						
Sí	0,67	0,21-2,12	0,501			
No	Ref.					
Malformación cardiaca						
Sí	1,31	0,61-2,81	0,480			
No	Ref.					
Comorbilidades						
Sí	2,87	1,40-5,9	0,004			
No	Ref.					
Alteración de sensorio						
Sí	0,92	0,34-2.50	0,884			
No	Ref.					
Dificultad respiratoria						
Sí	0,55	0,15-1,97	0,365			
No	Ref.					
Taquicardia						
Sí	0,75	0,29-1,89	0,547			
No	Ref.					
Taquipnea						
Sí	0,71	0,33-1,53	0,385			
No	Ref.					
Score PIRO modificado						
Leve	Ref.			Ref.		
Moderado	7,84	3,55-17,33	<0,001	3,40	1,46-7,93	0,004
Alto	17,00	8,66-33,36	<0,001	4,33	1,86-10,09	0,001
Saturación de oxígeno	0,99	0,90-1,09	0,906			
Proteína C reactiva(mg/L)	1,00	0,99-1,00	0,616			
Procalcitonina(ng/ml)	1,01	0,98-1,04	0,389			
Índice neutrófilos/linfocitos	1,03	0,91-1,18	0,554			
Hemocultivo						
Negativo	Ref.					
Positivo	2,19	0,97-4,97	0,058			
No tiene	0,46	0,17-1,22	0,120			
Compromiso multilobar en radiografía						
Sí	1,76	0,84-3,69	0,130			
No	Ref.					
Neumonía complicada						
Sí	10,57	4,79-23,33	<0,001	5,88	2,46-14,06	<0,001
No	Ref.			Ref.		

a Se realizó ajuste por variables edad, score PIRO modificado y neumonía complicada según criterio estadistico. No se incluyo la variable comorbilidades en el analisis multivariado porque es un parametro incluido en el score PIRO modificado por lo que existe colinealidad.

RPc: razón de prevalencias cruda, RPa: razón de prevalencias ajustada, UCI: unidad de cuidados intensivos.

PIRO: (P) Predisposition, (I) Insult, (R) Respond and (O) Organ dysfunction.

### Análisis multivariado de ingreso a cuidados intensivos

En el análisis multivariado, con respecto la edad, por cada mes adicional de vida, la probabilidad de ingreso a UCI disminuye en un 1% (RP: 0,99, p=0,011, IC95%: 0,98-0,99). Los pacientes con neumonía complicada tienen cinco veces la probabilidad de ingreso a UCI (PR=5,88, p<0,001, IC95%: 2,46‒14,06) que los pacientes con neumonía no complicada. Asimismo, los pacientes que presentaban un score PIRO modificado moderado tienen 3,4 veces la probabilidad de ingreso a UCI de los que tenían score PIRO modificado leve (RP=3,40, p=0,004, IC95%:1,46‒7,93) y los pacientes con score PIRO modificado alto tenían 4,33 veces la probabilidad de ingreso a UCI en comparación de los que tenían score PIRO modificado leve (RP= 4,33, p=0,001, IC95%:1,86- 10,09). En todos los casos, hubo una asociación estadísticamente significativa incluso luego del ajuste por variables como edad y neumonía complicada (Tabla 3).

## DISCUSIÓN

En este estudio se revisaron las historias clínicas de 166 pacientes hospitalizados con diagnóstico de neumonía adquirida en la comunidad en el INSNSB. La prevalencia de ingreso a UCI fue 14,5%. La menor edad, la presencia de neumonía complicada y la mayor gravedad evaluada con el puntaje PIRO modificado fueron variables asociadas al ingreso a cuidados intensivos.

La prevalencia de ingreso a UCI en el presente estudio (14,5%) fue menor a la reportada por Fritz *et al*, quienes encontraron que de los niños menores de 18 años hospitalizados por neumonía en tres hospitales de los Estados Unidos de Norteamérica enrolados en el estudio EPIC (Etiology of Pneumonia in the Community Study), el 21% de los que no tenían bacteriemia y el 43% de los que tuvieron bacteriemia ingresaron a cuidados intensivos ^11^. Sin embargo, en un estudio prospectivo con niños menores de cinco años ingresados por neumonía en un hospital de Vietnam, la prevalencia de admisión en cuidados intensivos fue menor (8%) ^12^. Con respecto al reporte en otros países de Latinoamérica, los resultados han sido similares al presente estudio; en un estudio transversal en niños menores de cinco años que tenía el objetivo de identificar los factores predisponentes para neumonía grave en niños hospitalizados en un hospital colombiano, se reportó una prevalencia de ingreso a UCI del 11,3% ^13^. Estas diferencias pueden deberse a varios factores, el nivel de complejidad de los hospitales ingresados en el estudio, la edad de los pacientes incluidos y a los criterios de ingreso a UCI en cada uno.

Este estudio encontró que, a menor edad, mayor es la prevalencia de ingreso a cuidados intensivos en niños con neumonía. De igual manera, otros estudios que han encontrado asociación entre menor edad del paciente y mayor prevalencia de resultados adversos como mortalidad. Así, Djelantik *et al*. describen que los pacientes menores de cuatro meses tuvieron mayor mortalidad en comparación a niños mayores (RR=3,5, IC95%: 3,0‒4,2) ^14^. Estos resultados se explican por factores anatómicos y fisiológicos que son más desfavorables a menor edad como la ausencia de canales de ventilación interalveolar y alveolo-bronquiolar que favorecen la aparición de complicaciones como atelectasias, a la mayor resistencia de las vías aéreas, menor compliance pulmonar y mayor compliance de la caja torácica en niños menores de dos años y a la inmadurez del sistema inmune innato y adquirido.

La neumonía complicada (efusión paraneumónica, empiema) se asocio a mayor probabilidad de ingreso a cuidados intensivos. Resultados similares a los de Golbart *et al*. quienes reportaron que la presencia de empiema se asocia al ingreso a UCI ^15^. Se ha reportado también que la presencia de efusión pleural moderada o grande se asocia con admisión a UCI en niños (OR: 3,2, IC95%: 1.1‒8,9) ^16^. Las afecciones ocupantes de espacio en la pleura pueden llevar a insuficiencia respiratoria por hipoventilación, lo que puede explicar la mayor necesidad de soporte ventilatorio en UCI que tienen estos pacientes.

El puntaje PIRO modificado discrimina la probabilidad de morir en niños hospitalizados con neumonía y puede ser una herramienta confiable para seleccionar pacientes que requieran admisión en cuidados intensivos ^17^^,^^18^. Este estudio encontró que los pacientes con puntaje PIRO modificado moderado y alto tenían más probabilidad de ingresar a UCI con respecto a los pacientes con puntaje PIRO leve. Estos resultados podrían ser un indicio de la utilidad de este puntaje para predecir el ingreso a UCI, sin embargo, para este estudio no se encontró información en la historia clínica de todos los ítems considerados en este puntaje como el valor de la presión arterial.

Este estudio no encontró asociación entre el compromiso multilobar en la radiografía de tórax y el ingreso a UCI. A diferencia de Williams DJ *et al*. que reportan que los infiltrados multilobares se asocian con resultados adversos graves como admisión a UCI, ventilación mecánica y muerte ^5^. Estos resultados discordantes podrían deberse a que existen factores que pueden alterar la interpretación de los resultados de la radiografía de tórax como el estado de hidratación, la presencia de atelectasias y el momento de presentación ^19^. En el presente estudio, los pacientes seleccionados estaban en el área de emergencia al ingreso hospitalario.

No se encontró asociación entre los niveles de INL con el ingreso a UCI. Este índice se valoró con el primer hemograma al ingreso del paciente donde probablemente no haya tenido antibiótico. Este resultado es diferente a los reportados por Lee *et al*. quienes encontraron que la elevación de INL estuvo asociada a mayor mortalidad y fue un predictor de ingreso a UCI en adultos, esta diferencia podría deberse al desarrollo inmunitario en los niños, en los cuales, predomina la inmunidad celular frente la inmunidad innata, lo cuál podría explicar que tengan mayor cantidad de linfocitos que neutrófilos en comparación de los adultos^20^.

Los pacientes con comorbilidades tuvieron mas probabilidad de ingreso a UCI, solo en el modelo crudo. Esto concuerda con los resultados publicados por Koh *et al*. quienes encontraron que la presencia de comorbilidades es una variable de pronóstico temprano para resultados desfavorables en pacientes que ingresaron a la unidad de cuidados intensivos ^21^.

Este estudio tiene limitaciones, al tener diseño retrospectivo, no se han evaluado algunas variables de interés como el estado vacunal contra patógenos causantes de neumonía, el estado nutricional o el tiempo que estuvo el paciente en emergencia antes de ir a UCI. Además, no se contó con el dato del valor de la presión arterial en todos los pacientes y la alteración de este valor es usado para construir el puntaje PIRO modificado. No existen criterios unificados para decidir el ingreso a UCI en niños con neumonía en el INSNSB. Además, el tamaño muestral es pequeño por lo que se tienen intervalos de confianza amplios y podría haber asociaciones reales no encontradas en el presente estudio.

En conclusión, la prevalencia de ingreso a cuidados intensivos en niños con neumonía fue de 14,5%. La menor edad, la presencia de neumonía complicada y la mayor gravedad evaluada con el puntaje PIRO modificado se asociaron al ingreso a UCI en niños con neumonía adquirida en la comunidad. Se recomienda aplicar PIRO modificado para predecir el ingreso a UCI en estudios prospectivos y con mayor tamaño muestral para mejorar precisión en los resultados.
